# A Diabetes Prevention Assessment Tool for American Indians

**Published:** 2005-09-15

**Authors:** Christopher A Taylor, Kathryn S Keim, Dale R Fuqua, Christine A Johnson

**Affiliations:** Medical Dietetics Division, The Ohio State University; Department of Clinical Nutrition, Rush University, Chicago, Ill; School of Educational Studies, Oklahoma State University, Stillwater, Okla; Bureau for Social Research, Oklahoma State University, Stillwater, Okla

## Abstract

**Introduction:**

American Indians have a disproportionately higher risk of developing type 2 diabetes. Few data are available about the perceptions of diabetes among American Indians, and no culturally appropriate tools are available for assessment of perceptions related to health and diabetes.

**Methods:**

A diabetes prevention assessment tool was developed to measure perceptions of health and diabetes among American Indians. Predominant themes from qualitative interviews were used to develop the items for the tool. Data were collected at two autumn powwows, or intertribal dances, in Oklahoma. Reliability testing was performed using 185 surveys from American Indian adults not living on reservations. Principal axis factor analysis was performed to identify possible relationships among the items.

**Results:**

Five themes, or factors, were found to categorize the perceptions of health: 1) lifestyles, 2) barriers to healthy lifestyles, 3) personal responsibility, 4) self-care behaviors, and 5) culturally defined well-being. Two factors classified the perceptions of diabetes: 1) a cognitive factor, related to personal experience, and 2) an affective factor, related to emotions.

**Conclusion:**

Our diabetes assessment tool identified factors that should be considered when developing health promotion and diabetes prevention programs for American Indians. A valid assessment tool for the American Indian population could provide valuable, formative data that would increase understanding of the culturally related obstacles to health promotion and diabetes prevention.

## Introduction

Diabetes has become one of the most prevalent chronic diseases in the United States; approximately 8.2% of the population has been diagnosed with the disease (1). Minority populations, especially American Indians, have a disproportionately higher rate of diabetes (2-5). Indian Health Service (IHS) data for the Oklahoma area (Oklahoma, Kansas, and a portion of Texas) — the IHS area with the most American Indians — have shown that the age-adjusted rate for diabetes is approximately 60 per 1000 individuals (4), indicating that American Indians are 2.43 times more likely to have diabetes than the general population (6).

Despite the clear impact of culture on health beliefs and lifestyle behaviors (7-10), limited data are available on the culturally related perceptions of health and diabetes among American Indians. Various researchers have studied the American Indian perceptions of health and diabetes through the lens of Western medicine (8-13). Their reports indicate that American Indian views of health and diabetes differ considerably from the central dogma of Western medicine, which classifies health in terms of physiologic symptoms (10-12). In one study, researchers found that Dinéalso known as Navajo) Indians placed little emphasis on the long-term effects of asthma, a chronic condition involving airway inflammation (10). Diné families who had children with asthma described asthma as a series of individual bouts of severe reactions requiring emergency medical attention (10). In other words, a child was thought to be cured of asthma if the child was not currently having reactions that required medical attention. Children often endured bouts of acute asthma with no medical treatment because families thought it would train the body to handle the condition. Similarly, our qualitative interviews of American Indian women (12) and men (C.A.T., unpublished data, 2002) in Oklahoma revealed that American Indians defined health by the presence or absence of physical symptoms of disease. In the absence of physical discomfort or limitations, they considered themselves to be healthy.

Some researchers have reported finding a sense of hopelessness and resignation among American Indians living on reservations about the inevitability of developing diabetes (8,14-16). From 1981 through 1983, Lang interviewed Dakota Sioux Indians to research the impact of diabetes on American Indian culture (13). Themes of disease pervasiveness, concerns for quality of life, and feelings of inevitability were found. Interviews with Pima (14) and Seminole American Indians (15) showed that they, too, felt developing diabetes was inevitable. Overall, the feelings of inevitability are a barrier to health promotion and diabetes prevention, especially when the physical cues in the form of diabetes symptoms are absent (9,12). The impact of these cultural perceptions on health is important. In several studies, the differences between American Indian health perceptions and Western ideology were not addressed, so the health promotion and diabetes prevention efforts among American Indians were not extremely effective (8,10,14).

Much of the research on health and diabetes perceptions among American Indians has been conducted in the reservation setting. Oklahoma does not have a reservation system and thus is a different research environment. A greater understanding of perceptions of health and diabetes among American Indians who do not live on reservations is paramount to the success of health promotion and diabetes prevention programs nationwide (7,17-20). Furthermore, the primary focus of existing tools for measuring diabetes perception is care of individuals who already have diabetes, not diabetes prevention.

A culturally appropriate instrument that measures perceptions of health and diabetes would provide helpful data for defining the relationship between perceptions and behavior. Understanding the relationship is critical for the development of targeted health promotion and diabetes prevention programs (21). If the onset of diabetes could be prevented or delayed, the improvements in quality of life and health care costs would be considerable (22-25).

## Methods

### Assessment tool development

In a previous study, 79 American Indian women (12) and 20 American Indian men (C.A.T., unpublished data, 2002) were interviewed to identify cultural perceptions of health and diabetes. We used qualitative data from the interviews to create items, or statements, for our assessment tool, which increased its content validity because it included issues more germane to the respondents (26). Using dominant themes and text from the interviews, we created the *Keeping the Balance Diabetes Assessment Tool,* a four-part questionnaire measuring diabetes knowledge and perceptions of health, diabetes, and the social environment with a focus on diabetes prevention.

We created an initial list of items, or statements, to address four major categories: 1) perceptions of health, 2) perceptions of diabetes, 3) knowledge about the etiology of diabetes, and 4) the role of social interactions in health maintenance. When creating the items for the questionnaire, we attempted to use the original wording of the respondents from the interviews (12) to increase content validity. Each item was a statement about health, diabetes, or the social environment and was measured using a 6-point Likert-type scale (with 1 indicating *strongly agree* and 6 indicating *strongly disagree*).

Experts with experience in questionnaire development, American Indian research, or American Indian clinical practice were recruited by personal invitation and through a research and an American Indian health care e-mail Listserv. Eleven experts responded to our request and volunteered to provide online feedback and rewording suggestions so that the items would accurately measure a single concept. The panel of experts reviewed the items for cultural appropriateness, clarity, conciseness, and the ability to measure the intended concept. We used the panel's comments to create the final version of the instrument.

### Sample and data collection

Men and women aged 18 to 65 years who were at least 25% American Indian according to a self-report were eligible to participate. Individuals were not excluded if they had diabetes or other chronic diseases. Key informants at two tribal health clinics identified two powwows in northeast Oklahoma where we could use the assessment tool. After receiving invitations to the powwows, a research team attended them in September 2003 and collected data.

At the first powwow, 81 volunteers completed the assessment tool; 116 volunteers completed the assessment tool at the second powwow. Two participants were excluded because they did not meet study criteria. Ten participants were excluded after providing incomplete responses or abnormally patterned responses (e.g., choosing all *A*'s for every answer). Using SPSS (SPSS Inc, Chicago, Ill), data analysis was performed using 185 (94%) of the 197 completed questionnaires. The Oklahoma State University Institutional Review Board approved the study protocol.

Each volunteer provided signed informed consent before participating. The self-administered questionnaire was presented to eligible volunteers and followed by a brief demographic questionnaire. To increase participation, volunteers who completed both questionnaires received $10.

### Data analysis

Items from two scales (31 health perceptions items and 21 diabetes perceptions items) were analyzed using principal axis factor analysis. Factor analysis is used to assess item correlations and identify common relationships among similar items, allowing the items to be categorized into various themes, or factors (27). The resulting factors are named based on the overall theme of their corresponding items. Data were excluded pairwise for items with missing or multiple responses. Data from the social interaction scale are not discussed in this article.

The principal factor analysis for each scale involved a standardized approach, and each scale (health and diabetes) was analyzed independently. The correlation matrix, Kaiser-Meyer-Olkin (KMO) measures of sampling adequacy, and Bartlett's tests of sphericity were evaluated for the factorability of the correlation matrix (i.e., to determine whether the items could indeed be classified into a few categories) (27). KMO values greater than 0.6 indicated that the correlation matrix had sufficient structure to result in a factorable solution. A significant Bartlett's test of sphericity indicated that the correlation matrix was significantly different from an identity matrix (a correlation matrix in which items correlate perfectly with themselves and not at all with other items).

To clarify the factor pattern, a rotation analysis of the factors was performed. The eigenvalues (measures of variance) greater than 1 in conjunction with the scree plot (a plot of the eigenvalues and the factors) were assessed to determine the number of factors to use in the rotation analysis (27). The analysis was performed with an oblique rotation (direct oblimin, Δ = 0), which allows the factors to correlate. The correlations between the linear factors were evaluated and if little correlation resulted, the analysis was repeated using an orthogonal (varimax) rotation. Items accounting for at least 16% of the variance on a factor (with loadings greater than an absolute value of 0.4) were considered to *load,* or be sufficiently correlated with, a particular factor (27). All items loading on a factor were then used as the basis for naming the factor. Cronbach α was computed for all items loading on each factor. Values higher than an absolute value of 0.7 were considered to have acceptable internal consistency (28).

## Results

At the two powwows, 197 volunteers completed the assessment tool. Data analysis was performed on 185 questionnaires. Approximately two thirds of the volunteers were female, with a mean age of 37 years and a mean of 69% American Indian ancestry. Of the 185 participants, 48 (26%) reported having had a previous diagnosis of diabetes.

### Factor analysis: health perceptions

Evaluation of the correlation matrix indicated relationships among the items. The KMO (0.71) and Bartlett's test of sphericity (*P* <.001) indicated a factorable correlation matrix. The initial factors extracted from the 31 items measuring the health perceptions indicated a potential for extracting three, four, or five overall factors using the scree plot (Figure) and the eigenvalues (Table 1). We found that five factors best categorized the perceptions of health, which accounted for 33.1% of the total variance. An oblique rotation resulted in little correlation among the factors, so a varimax rotation was performed and interpreted.

**Figure:**
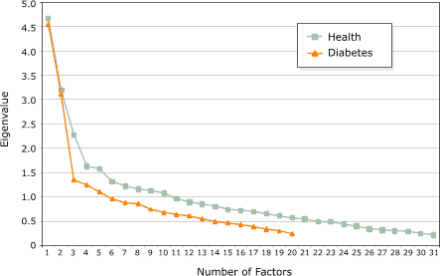
Scree plots with results from factor analyses of health and diabetes perceptions.

Items with factor loadings greater than or equal to an absolute value of 0.4 further clarified the factor's theme. The factor loadings for each of the five factors are presented in Table 2. The five items classified by the first factor (lifestyles) were related to health maintenance behaviors, whereas the second factor (barriers) comprised five obstacles to maintaining good health. Culturally related personal responsibility for health and wellness characterized the third factor (personal responsibility). The fourth factor was characterized by self-care behaviors and their association with health, and the fifth factor (cultural wellness) included items related to the association between health and mental and physical well-being.

Cronbach α internal consistency coefficients (Table 1) were modest for the five factors of health perceptions.

### Factor analysis: diabetes perceptions

Analysis of the correlation matrix, KMO (0.79), and Bartlett's test of sphericity (*P* <.001) for the 21 items related to diabetes perceptions suggested that the correlation matrix was factorable. Examination of the initial eigenvalues (Table 1) and the scree plot (Figure) suggested that two factors would best categorize the 21 items. A varimax rotation was performed because the oblique rotation resulted in weak correlations between the two factors. Two factors explained 32% of the variance of the diabetes perceptions. Factor loadings for both factors are provided in Table 2. Nine items related to knowledge of and personal experience with diabetes loaded on the first factor (cognitive). The second factor (affective) comprised eight items related to concerns about and fear of diabetes and its complications. One item, the perception of diabetes as a death sentence, loaded on both factors: cognitive (0.45) and affective (0.44). Because this item related to personal experience but also caused an emotional response, it was allowed to load on both factors.

Cronbach α internal consistency coefficients (Table 1) were desirable for the two factors of diabetes perceptions.

## Discussion

Understanding culturally related health factors is critical for health promotion and diabetes prevention efforts among minority populations (8,9,29,30). The paucity of data on health perceptions of American Indians who do not live on reservations prompted our previous interviews (12); our technique of using responses from interviews to create an assessment tool has also been used in research on health perceptions about type 2 diabetes among African Americans (31-35).

### Health perceptions

Of the factors identified for the health perceptions items, we discovered that two factors (lifestyle behaviors and self-care) involved the impact of behavior on health, a theme found in previous research studies (15,29). In our previous qualitative interviews, we found that participants believed that overall good health was related to a healthy lifestyle, even though they also felt that developing diabetes was inevitable (12). Responses to the lifestyle questions accounted for the greatest amount of variance (9.5%) in the health perceptions items in the assessment tool, indicating that respondents believed healthy lifestyle behaviors were essential for maintaining good health. In addition, the self-care factor had items related to the importance of taking an active role in health maintenance. Hatton (11) reported similar themes among urban American Indians, who believed that health maintenance was directly related to performing certain self-care tasks. In our study, the barriers factor comprised perceived difficulty in changing lifestyle behaviors and the perceived financial and time investments required for health maintenance, which conflict with the beliefs that self-care behaviors affect health.

Another dominant theme from our previous interviews was that the respondents relied on physical symptoms as indicators of their health status (12). Participants believed that their behaviors did not need to change if they had no physical symptoms of illness. The personal responsibility factor and self-care factor in this study included similar items, such as those measuring the cues to change lifestyle behaviors and variables used to define personal health status. The perception that a person is healthy if the person does not have a disease (a culturally defined definition of wellness) likely affects health promotion and diabetes prevention efforts.

### Diabetes perceptions

Racial and ethnic minorities are a medically underserved population in the United States (9,36). The differences in health status between American Indians and the general U.S. population are becoming more pronounced as rates of various chronic diseases, including diabetes, begin reaching epidemic levels in American Indians (37). In some southwestern American Indian tribes, as many as 50% of adults older than age 35 years are affected (22). The high diabetes rate and its long-term complications (38-41) likely shape the cognitive and affective factors in the assessment tool items measuring diabetes perceptions.

Strong feelings of hopelessness and fear (affective factors) related to the effects of diabetes were evident during our previous interviews (12) and in research with other minority groups (14-16,42). In fact, Arizona Pima Indians have developed a cultural defense mechanism now known as *surrender,* which stems from the perceived futility of diabetes prevention (14) and hinders the initiation of diabetes prevention behaviors.

The items that loaded on the cognitive factor likely reflect diabetes knowledge obtained from personal experience. In another study, personal experiences of individual Dakota Sioux Indians collectively shaped an entire tribe's cultural perceptions of health and diabetes (13). Experience has also shaped the attitudes of Mexican Americans about health and illness and has affected their social functioning and physical and mental health (42). Our previous interviews revealed that the personal experiences of American Indians with diabetes shaped their perceptions of diabetes prevention, treatment, and etiology (12).

Even though they recognized the relationship between healthy lifestyles and good health, participants in our study believed that their lifestyle behaviors did not have to change until their diabetes resulted in perceivable signs and symptoms. Likewise, in another study, researchers found that African American women did not consider diabetes to be a serious disease and thought medication alone was the cure (43). These findings were similar to findings from our study; participants believed that diabetes treatment involved only medication if they had no diabetes symptoms.

Items measuring the susceptibility of American Indians to developing diabetes and the fear of diabetes and its long-term complications characterized the affective factor. In a similar study involving Mexican Americans, researchers found that women judged the severity of diabetes by the extent of resulting physiologic damage (42). Diabetes was a frightening disease because of the extensive damage it could cause. Likewise, American Indian women thought of diabetes in terms of its complications, such as kidney failure and blindness (12). African American women thought that a lack of physical symptoms indicated they no longer had diabetes (43), but concerns about diabetes were strongly linked to emotional factors, including fears about diabetes, denial, and concerns about insulin therapy and required lifestyle changes (44).

### Limitations

Our study has several limitations. The sample was derived through nonprobability methods and may have decreased the generalizability of the findings, but these sampling methods are often needed to identify individuals from an at-risk population (11). The smaller sample size could account for the lower internal consistencies for some of the health factors and the loading of the "death sentence" item on the cognitive and affective diabetes factors. In addition, in future versions of the assessment tool, the wording of the items may need to be changed.

As mentioned, the internal consistency coefficients for three of the health perceptions factors (Cronbach α = 0.50–0.55) were lower than desired, possibly because of the partialing of variance — and the factoring of residualized variance — inherent in the factor analysis method or the smaller sample size. Furthermore, the sample size may not have been large enough to account for the variance associated with the health and diabetes perceptions. Methodologically, complications arose as we tried to determine the number of factors to rotate for health perceptions. Despite the lower Cronbach α values, the five factors provided a sound theoretical explanation of health perceptions. Additional testing of the assessment tool in larger samples is needed to determine the relevance of the items and the stability of the factor structure.

### Implications for the future

Previous studies have identified several health-related beliefs that seem to be common among various tribes: 1) the perception that health is the responsibility of the individual, 2) the perception that health and disease are natural parts of life, and 3) the understanding that spirituality plays a role in health (29). However, identifying differences among tribes and the extent to which individuals subscribe to their tribe's cultural beliefs are important when developing health promotion activities (9,29,30,45). This assessment tool could help clinical and public health professionals obtain data about health and diabetes perceptions among individuals or groups. The resulting data from this study could provide important information for health professionals who are attempting to create culturally appropriate health care counseling and health education programs, which should be customized for specific groups and designed according to the variables that influence the group's behavior (7,46-48).

As mentioned, we found similarities between our research and the findings from other studies, all of which demonstrate relationships among the factors associated with health and diabetes perceptions. Future research must expand the exploration of health and diabetes perceptions so that health professionals can design successful health promotion and diabetes prevention programs.

## Figures and Tables

**Table 1:** Initial Eigenvalues, Percentage of Variance, and Internal Consistencies for Perceptions of Health and Diabetes Factors Among American Indians in Oklahoma

**Factors**	**Eigenvalue**	**Cumulative Percentage of Variance**	**Cronbach ** **α**

**Health perceptions**			

Lifestyles	4.67	9.5	0.794
Barriers	3.20	18.0	0.756
Personal responsibility	2.29	23.4	0.500
Self-care	1.63	28.6	0.549
Cultural wellness	1.58	33.1	0.527

**Diabetes perceptions**			

Cognitive	4.54	17.9	0.831
Affective	3.12	32.0	0.773

**Table 2:** Factor Loadings on Perceptions of Health and Diabetes Among American Indians in Oklahoma

**Factors**	**Description**	**Loading**	**Mean (SD)^a^**

**Health perceptions**			

Lifestyles	Start early to take care of health.	0.787	1.8 (1.0)
	Start early to eat healthy.	0.744	2.0 (1.1)
	Start early to be physically active.	0.736	1.9 (1.0)
	Starchy foods are bad.	0.532	2.6 (1.3)
	A lot of fat is bad.	0.489	2.1 (1.2)
Barriers	It is hard to change your diet.	0.740	3.3 (1.5)
	It is hard to be more active.	0.669	3.5 (1.4)
	It is hard to eat healthy.	0.579	3.5 (1.4)
	Good health takes money.	0.539	3.4 (1.6)
	Good health takes time.	0.497	3.1 (1.5)
Personal responsibility	I go to the doctor when I feel sick.	0.532	3.5 (1.6)
	My heath shouldn’t burden others.	0.498	2.2 (1.1)
	Only I can take care of my health.	0.471	2.0 (1.3)
	I take care of my health when I feel sick.	0.459	2.9 (1.6)
Self-care	I prevent problems when I care for my health.	0.660	1.6 (0.9)
	I will be healthy if I take care of myself.	0.631	1.6 (0.8)
	I will stay healthy if I visit the doctor regularly.	0.405	2.3 (1.3)
Cultural wellness	Unhealthy people are irritable.	0.554	3.1 (1.5)
	Unhealthy people are depressed.	0.547	3.0 (1.4)
	I am healthy if I do not have a disease.	0.413	2.4 (1.4)

**Diabetes perceptions**			

Cognitive	I can control diabetes by medicine without changing my diet.	0.712	3.7 (1.6)
	I can control diabetes by medicine without changing my physical activity level.	0.673	3.7 (1.5)
	My behavior doesn't need to change until I get diabetes.	0.656	4.1 (1.4)
	I avoid screening so that I will not have to treat my diabetes.	0.630	4.5 (1.4)
	Diabetes is taking insulin shots.	0.593	3.5 (1.5)
	Diabetes is inevitable for American Indians.	0.583	4.6 (1.4)
	You can tell that someone has diabetes by looking at them.	0.540	4.7 (1.4)
	I won’t think about diabetes until it happens to me.	0.400	4.2 (1.5)
	Diabetes is a death sentence.	0.450	3.6 (1.6)
Affective	Diabetes is scary.	0.679	2.2 (1.3)
	Diabetes ruins health.	0.602	3.0 (1.4)
	I am afraid of diabetes.	0.583	2.7 (1.5)
	Diabetes requires a lot of changes.	0.529	2.6 (1.4)
	Diabetes attacks your organs.	0.491	2.5 (1.3)
	People with diabetes need to eat different foods.	0.511	2.7 (1.4)
	American Indians are at higher risk for getting diabetes.	0.467	1.9 (1.2)
	Diabetes is a death sentence.	0.439	3.6 (1.6)

a Responses according to a 6-point Likert-type scale. 1 indicates strongly agree; 2, agree; 3, somewhat agree; 4, somewhat disagree; 5, disagree; and 6, strongly disagree.
